# Molecular Detection and Drug Resistance of *Mycobacterium tuberculosis* Complex from Cattle at a Dairy Farm in the Nkonkobe Region of South Africa: A Pilot Study

**DOI:** 10.3390/ijerph9062045

**Published:** 2012-05-29

**Authors:** Blessing Silaigwana, Ezekiel Green, Roland N. Ndip

**Affiliations:** 1 Department of Biochemistry and Microbiology, University of Fort Hare, P.O. Box X1314, Alice 5700, South Africa; Email: silaigwana@yahoo.com (B.S.); rndip@ufh.ac.za (R.N.N.); 2 Department of Microbiology and Parasitology, University of Buea, P.O. Box 65, Buea, Cameroon

**Keywords:** *Mycobacterium tuberculosis* complex, unpasteurized milk, PCR, multidrug resistance, South Africa

## Abstract

*Mycobacterium tuberculosis* complex (MTBC) causes tuberculosis (TB) in humans and animals. We investigated the presence of MTBC in cattle milk and its drug resistance using polymerase chain reaction (PCR). Two hundred samples (100 mL each) were obtained from a dairy farm in the Nkonkobe region of South Africa. The samples were processed using the modified Petroff method. DNA was isolated using a Zymo Bacterial DNA kit and amplified using Seeplex^®^ MTB Nested ACE assay. The Genotype^®^* Mycobacterium tuberculosis*-multidrug resistant*plus* (MTBDR*plus*) assay was used to perform drug susceptibility and detection of mutations conferring resistance to isoniazid (INH) and rifampicin (RIF). Eleven samples tested positive for MTBC DNA using the Seeplex^®^ MTB Nested ACE assay. The Genotype^®^ MTBDR*plus* assay showed that 10/11 samples were resistant to both INH and RIF *i.e.*, multi-drug resistant (MDR). The most and least frequent *rpoB* mutations detected in RIF resistant samples were H526Y (9/10) and D516V (2/10) respectively. None of the INH resistant samples harbored mutations in the *katG* gene. However, all of them harbored the T8A mutation in the *inhA* gene. These results have clinical and epidemiological significance and calls for further studies and necessary actions to delineate the situation.

## 1. Introduction

Bovine tuberculosis (BTB), a prominent disease found in cattle, is mainly caused by *Mycobacterium bovis*, a member of the *Mycobacterium tuberculosis* complex (MTBC) which includes *M. tuberculosis*, *M. africanum*, *M. bovis* BCG, *M. canetti*, *M. microti*, *M. caprae* and *M. pinnipedii* [1]. BTB has been reported in South Africa since 1880 and *M. bovis* strains have been isolated from cattle in most of the country’s provinces [2,3]. The introduction of the national BTB control and eradication scheme in 1969 resulted in a sharp decline in the prevalence of the disease [2]. However, between 1992 and 1998, there was a dramatic increase in the prevalence of BTB up from 4.4 and 27.1% to 16 and 38.2% in the central and south regions respectively [4]. Spillover of infection at the wildlife-livestock interface has been implicated as a significant risk factor for the increase of BTB in South Africa [3,4].

BTB remains a worldwide problem, with more than 50 million cattle estimated to be infected [5]. The disease is an important zoonosis which poses a significant threat to humans as it can be transmitted through consumption of contaminated milk and close contact with infected cattle [6]. *M. bovis* accounts for approximately 0.5–1.5% of the entire human TB cases in some developed countries [7]. Similar studies reported a higher prevalence (5–10%) of human *M. bovis* infection in developing countries, which has been attributed to poor BTB control in these countries [8]. Approximately 85% of cattle and 82% of the human population in Africa reside in areas where there is a high prevalence of BTB [9,10]. The precise involvement of *M.bovis* to the global burden of human TB remains ambiguous because *M.tuberculosis* and *M. bovis* are clinically indistinguishable [9]. Moreover, differentiation of MTBC using conventional methods and biochemical tests is laborious and time consuming [10]. *M. bovis* is naturally resistant to pyrazinamide (PZA), a first line anti-TB drug; therefore accurate identification of the source of infection is important for patient management and control [9]. Effective control measures such as test and slaughter strategy and pasteurization of milk have greatly contributed to the reduction of BTB in most developed countries [11]. Sadly, human TB due to *M. bovis* infection remains heavily skewed towards developing countries where control strategies are neglected or do not exist at all [9].

The emergence of resistance to key TB drugs is associated with low cure rates and high mortality, thus, greatly impeding efforts to control the epidemic [12]. This underscores the need for novel diagnostics allowing rapid identification of drug resistant strains in order to initiate early treatment and circumvent further transmission. MDR strains of TB, *i.e.*, resistant to at least INH and RIF has been reported worldwide, with approximately 500,000 cases reported in 2007 [13]. Approximately 5–7% of MDR cases subsequently develop into extensively drug resistant (XDR) form of TB [14]. XDR-TB, *i.e.*, resistance to either INH or RIF in addition to any member of the quinolones and at least one injectable anti-TB drugs kanamycin, capreomycin and amikacin has reportedly been confirmed in at least 58 countries worldwide, including South Africa [13,15]. Mutations in the rifampicin resistance determining region (RRDR) of the *rpoB* gene confer resistance to RIF, an important first-line drug [12,16]. Resistance to RIF can serve as surrogate marker for MDR-TB since approximately 90% of RIF resistant strains are likewise INH resistant [17]. Resistance to INH occurs as a result of different mutations in various genes namely *katG*, *inhA*, *kasA*, *ahpC* and *oxyR* [16,18]. Mutations on codon 315 of the *katG* gene are often the most frequent accounting for approximately 95% of INH resistance in *M. tuberculosis* whereas mutations in the *inhA* gene confers resistance in approximately 35% cases [16]. The development of new molecular methods based on PCR and sequencing in recent years, has allowed the rapid identification and detection of genetic mutations and single nucleotide polymorphism related to resistance in *M. tuberculosis* strains. From these PCR based methods, easy to use commercial kits such as the INNO-LiPA Rif.TB (Innogenetics, Ghent, Belgium) and Genotype^® ^MTBDR*plus* (Hain Lifescience, Nehren, Germany) have been developed [18].

There is a dearth of information on the prevalence and drug susceptibility of MTBC circulating in dairy cattle in the Nkonkobe region. This would assist in the management and control of MTBC related infections; hence, this study aimed to use molecular techniques for the detection and drug susceptibility testing of MTBC in unpasteurized milk at a dairy farm in the Nkonkobe region of the Eastern Cape Province.

## 2. Results and Discussion

### 2.1. Prevalence of MTBC amongst Different Cattle Breeds

MTBC was detected in all the four breeds of dairy cattle used in this study. A summary of the characteristics of all the 11 cows that tested positive for MTBC are shown in Table 1. The frequency of MTBC varied; each breed and age group showed a particular prevalence. Overall, the Friesland breed had 4/50 cows with detectable MTBC. Few MTBC were detected in the Guernesey (2/50) and Aryshire (2/50) breed.

**Table 1:** Characteristics of cows that tested positive for MTBC.

Total herd	Breed	Age	Sex	Body condition	TB history	Vaccinated against BTB	Number positive for MTBC	Overall positivity per breed
50	Jersey	<3 years	F	Good	Unknown	No	1	3
		>3 years	F	Good	Unknown	No	2	
50	Friesland	<3 years	F	Good	Unknown	No	1	4
		>3 years	F	Good	Unknown	No	3	
50	Guernsey	<3 years	F	Good	Unknown	No	1	2
		>3 years	F	Good	Unknown	No	1	
50	Ayrshire	<3 years	F	Good	Unknown	No	1	2
		>3 years	F	Good	Unknown	No	1	
Total (200)								11

F, female.

### 2.2. Amplification of DNA Samples

**Figure 1:**
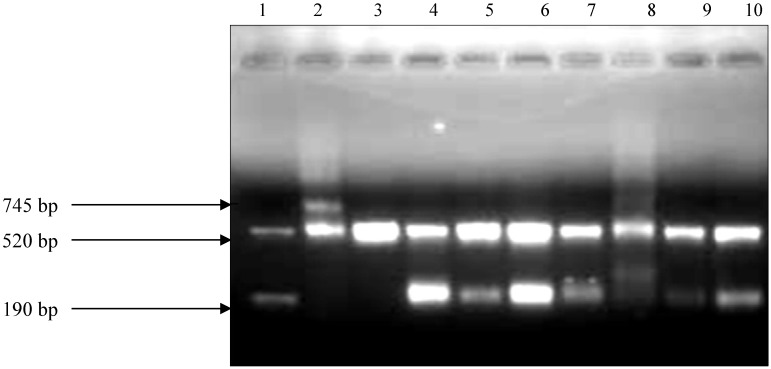
PCR results of the MTB Nested ACE detection assay followed by electrophoresis of the amplicons on 2% agarose gel. A negative sample shows only the internal control band (520 bp), while positive samples shows internal control and the *M. tuberculosis* band corresponding to 190 bp. The positive control does not have a band at 190 bp, instead it contains an upper band corresponding to 745 bp. According to the manufacturer, this is designed to eliminate false positivity resulting from cross contamination. Lane 1, DNA Marke (shows how a postive sample should be); Lane 2, positive control; Lane 3, negative control; Lane 4–9, Positive milk samples, Lane 10, H37Rv control strain.

The Seeplex^® ^MTB Nested ACE detection assay (Seegene Inc., Seoul, Korea) was used (Figure 1). The assay uses multi target (*mpb*64 and IS*6110*) PCR instead of a single target PCR for specific detection of MTBC. The internal control (520 bp) is used to identify processed samples containing substances that may interfere with PCR amplification.

### 2.3.Drug Susceptibility Results

The GenoType^®^ MTBDR*plus* assay showed that of the 11 positive samples, only one was sensitive to both INH and RIF, whereas a total of 10 were resistant to both INH and RIF (Table 2). The samples that were detected as MDR were from all the four breeds used in the study. The sensitivity of the assay was 90.9% (10/11) whereas specificity was 100% for both INH and RIF resistance. 

**Table 2:** Drug susceptibility pattern of the samples.

Antibiotic Susceptibility pattern		Genotype MTBDR *plus* assay	
		Number of samples	
RIF	Resistant	10	
INH	Resistant	10	
RIF & INH	MDR	10	
RIF & INH	Sensitive	1	

MDR, multi-drug resistant; RIF, rifampicin; INH, isoniazid.

### 2.4. Mutations Conferring INH and RIF Resistance

Of the 10 samples identified as RIF resistant using the GenoType^®^ MTBDR*plus* assay, nine harboured H526Y mutations, whilst only two samples possessed D516V mutations in the *rpoB* gene. None of the INH resistant samples harboured detectable mutations in the *katG* gene. All 10 samples showing INH resistance harboured T8A mutations in the *inhA* gene (Table 3).

**Table 3:** Frequency of mutations conferring INH and/or RIF resistance.

Mutation probe	Codon analyzed	Type of mutation	Number of samples
***rpoB* (RIF resistance )**			
MUT1	513–519	D516V	2
MUT2A	526–529	H526Y	9
MUT2B	526–529	H526D	8
MUT3	530–533	S531L	6
***katG* (High level INH resistance)**			
MUT1	315	S315T1	0
MUT2	315	S315T2	0
***inhA* (Low level INH resistance)**			
MUT1	–15	C15T	6
MUT2	–16	A16G	6
MUT3A	–8	T8C	6
MUT3B	–8	T8A	10

The need to implement sanitary guidelines so as to guarantee public health has been highlighted by an increase in MTBC infections concomitant with consumption of unpasteurized milk, home-made dairy products and food handling reported in the World [6]. More importantly, lack of information on the TB history of all the cattle in our study raises concern on the public health implication on humans interacting with those cattle as well as consumption of unpasteurized milk from the herd. Differential diagnosis should take priority in control plans to enable the optimal use of veterinary interventions as a means to reduce the burden of human disease from an animal source. In this study, we used the Seeplex^® ^MTB Nested ACE assay, a multiplex PCR system for the detection of *M. tuberculosis* in clinical specimens such as sputum, body fluids, bronchial washings, tissues, urine and stools. We found out that it can also effectively be used on milk samples; hence could be a useful alternative to the laborious tuberculin skin test (TST) and culture method for laboratory diagnosis of BTB in cattle. This method is easy and fast, yielding results in less than seven hours from the time of sample collection. However, it is expensive; hence making it difficult for routine diagnosis of TB in resource limited settings. 

Our results indicate that 11/200 samples tested positive for MTBC. These results are comparable with other reports whereby MTBC has been detected in 36/50, 7/68 and 47/54 milk samples screened in the respective studies [6,10,19]. In another similar study aimed to characterize MTBC in cattle using PCR, the authors reported that they isolated 43 MTBC isolates from 1,067 bovine throat swab samples obtained from cattle that had shown positive results of TST [1]. Although we did not culture the bacteria as well as perform TST to determine the number of cows positive for MTBC, our study showed a high sensitivity and specificity of the PCR assay for the detection of MTBC in cow milk. Therefore, this may be used as a rapid alternative to the time-consuming culture method and less specific TST for diagnosis of BTB in cattle. The results from our study and those obtained by other researchers certainly indicate that TB in cattle remains a widespread problem in both developing and some developed countries. The discrepancies in prevalence of MTBC may be related to different sample sizes used in the various studies as well as in different control strategies implemented in these countries [9]. 

It is interesting to note that MTBC was detected in all the four breeds of cattle investigated in this study, albeit there was a subtle difference in the prevalence within each breed. This could be an indication that BTB can infect any cow exposed to MTBC in one way or another, despite its genetic makeup (breed) or age group. However, other authors reported that age and breed are some of the risk factors associated with BTB infection in cattle. Older cattle are at more risk of BTB infection than young calves possibly because the duration of exposure is likely to increase with age [20,21]. Risk factors for BTB in cattle vary with countries and they can have their origin at the animal-level, e.g., breed and age or at the herd-level, e.g., herd size or history of BTB outbreak in the herd [20]. In Uganda, the disease is associated with different types of drinking water sources and areas of production, whilst in Eritrea, BTB is associated with communal grazing, management system and the animal breed [22]. Several studies have shown that exotic breeds are likely to be more infected with BTB than indigenous breeds [20]. However, some studies in Tanzania contradicts this hypothesis as they reported that the indigenous Zebu breed reacted more positively to the TST compared to the exotic breeds [21]. Although the specific risk factors for BTB in South Africa remains unclear, spillover from wildlife particularly the African buffalo in the country’s game parks has been identified as a risk factor for infection in communal cattle herds [4].

Another fascinating observation made in this study is that although all the cows had a good body condition (no observable clinical signs of the disease), results of the PCR assay showed that their milk was infected with MTBC. This could be explained by the fact that cattle with BTB may not show any clinical signs even during the advanced stages of the disease [6]. Furthermore, the signs may not be noticed by the veterinarians or cattle owners because of negligence. Therefore, the reduction of BTB in cattle could be achieved through vaccination using the *M. bovis* BCG strain since there are no other vaccines available on the market.

Although the Seeplex^®^ MTB Nested assay could not ascertain the MTBC organisms detected in the milk samples, various assumptions have been made. Cattle are always in close contact with dairy farm workers and other people who may have TB; there is the possibility of transmission of *M. tuberculosis* from an infected person to cattle [7]. Therefore we postulate that the DNA could represent *M. tuberculosis*. Various studies have reported the isolation of *M. tuberculosis* in cow milk [23,24]. However, since *M. bovis* is the predominant MTBC pathogen in cattle, we also speculate that the DNA could represent *M. bovis.* Cattle infected with BTB shed *M. bovis* in their milk and this has been previously reported [23,25]. Furthermore, we think that the detected DNA could represent other MTBC organisms, although little is known about their ability to infect cattle. We suggest that the high prevalence of MTBC in our present study could result from co-habitation with humans who may have active TB, hence transmit bacteria to cattle through aerosols. Another possibility is that cattle already infected with TB may transmit the bacteria to healthy cows through sharing of a common water source. Furthermore, cattle with BTB shed bacteria via urine and other excreta, hence other cows grazing on the contaminated grass may ingest or inhale MTBC. Additionally, calves are fed using milk from adult cows, therefore there is a possibility of infection through consumption of milk infected with MTBC. Since BTB is chronic, the cows may grow up having the disease without showing any clinical signs [6].

We found a high prevalence of MDR in the cattle screened in our study (10/11 samples). Only one sample was susceptible to both RIF and INH. Our results are comparable with those obtained in a similar study in Italy where 14/22 INH and/or RIF resistant strains of *M. bovis* isolates in cattle were reported [26]. Of the 14 resistant strains, five were characterized as MDR. To the best of our knowledge, no cases of MDR in MTBC obtained from cattle have been reported in Nkonkobe Region, South Africa. We therefore present the first report on MDR in cattle in the Nkonkobe region. 

The high prevalence of MDR in the study presents a health risk to local villagers who depend on unpasteurized milk for consumption. It is interesting to observe such a high level of drug resistance in cattle since animals are not treated for TB. We therefore postulate that the MDR strains detected in cattle screened in our study could have their origin from a human source because of non-compliance or inadequate TB treatment of the patients. Hence, the strains may have been transmitted to the cattle through close contact with farm workers or herdsmen infected with MDR-TB. Our study could have been improved by gathering information such as the TB status of the farm workers, their TB history and whether or not they have been treated for the disease previously. This would have helped to ascertain if the source of MDR in the cattle originated from the farm workers. 

Genotypic analysis of the RIF resistant samples indicated four different nucleotide substitution mutations involving codons 516, 526 and 531 of the *rpoB* gene. The H526Y mutation was identified in nine out of 10 RIF resistant samples, thus rendering it the most frequent mutation conferring RIF resistance in this study. This was followed by the H526D mutation encountered in eight out of 10 samples. Our results are different from those obtained by Sechi *et al.* [26] where L521P mutation were reported in six of 10 RIF resistant *M. bovis* isolates from cattle. Additional mutations identified in RIF resistant samples in our study were S531L (6/10 samples) and D516V (2/10 samples). Our results are different from other authors as no S531L or D516V mutation in RIF resistant *M. bovis* was reported [26]. This could possibly be due to the differences in the samples used and also the geographical location of the MTBC isolates analyzed in each study. 

With regards to INH resistance, we report that no mutations were identified in the *katG* gene of the samples used in this study. This could be explained by the fact that the Genotype MTBDR*plus* assay only detects mutations in codon 315 of the *katG* gene; hence additional mutations other than those occurring on codon 315 could not be detected. Our results are in contrast with the findings by Sechi *et al.* [26], who reported that two of the nine INH resistant *M. bovis* in their study harbored the S315T mutation in the *katG* gene. All INH resistant samples in this study harbored mutations in the *inh*A gene. The nucleotide substitution T8A was the most frequent mutation identified in all the INH resistant samples. We made an interesting observation in six of the 10 samples which had the T8A mutation. These samples tested positive for all the *inhA* wild type probes and their respective mutation probes, hence indicating the possibility of heteroresistance to INH. Heteroresistance results from the co-existence of susceptible and resistant *M. tuberculosis* strains and it has been reported in various studies [27,28].

The results of our study showed that molecular methods for detection of MTBC and drug resistance can be applied directly on milk samples without the need for culturing, which is time consuming and laborious. The main strength of the Seeplex^®^ MTB Nested ACE detection assay is that it uses multi-target PCR (IS*6110* and *mpb*64) for the specific detection of MTBC only, thereby preventing false positivity caused by other mycobacteria. However, the weakness of the assay is that it does not differentiate amongst members of the MTBC. The weakness of the Genotype^®^ MTBDR*plus* assay is that it only detected mutations in the *rpoB*, *katG* and *inhA* genes; therefore, resistance to INH and RIF caused by mutations in other genes could not be detected in this study. The results of our study highlight the need for effective control measures of BTB since it poses a significant risk to public health due to its zoonotic potential.

## 3. Experimental Section

### 3.1. Sampling

Four different breeds of cattle at a dairy farm were screened. Each breed comprised 50 cows (herd) with varying ages and health status. A total of 200 unpasteurized milk samples (100 mL each) were collected for the study. The samples were collected into sterile bottles. All the samples were placed in a cooler bag with ice and transported to the laboratory for analysis. 

### 3.2. Decontamination of the Samples

All the specimens were processed and decontaminated using the modified Petroff method [10]. Briefly, 10 mL of each milk sample were added into 50 mL centrifuge tubes and mixed with an equal volume of 7% NaCl solution and 4% NaOH solution. The mixture was vortexed for 15–20 s and then incubated in a water bath at 37 °C for 20 min. The decontaminated specimens were washed by adding sterile distilled water. The tubes were centrifuged for 15 min at 3,000 × g and the remaining pellet used for DNA extraction.

### 3.3. DNA Extraction and Amplification

DNA extraction and purification was performed using the Zymo research bacterial DNA kit (ZR Fungal/Bacterial DNA Kit^TM^, Inqaba Biotec, Pretoria, South Africa) according to the manufacturer’s instructions. Amplification of bacterial DNA was done using the Seeplex^®^ MTB Nested ACE detection assay (Seegene Inc, Korea) according to the manufacturer’s instructions using a thermal cycler (Bio-Rad, Cape Town, South Africa). The assay is a multiplex PCR system for the detection of MTBC. The amplification protocol involved the first PCR (1 cycle at 94 °C for 15 min; 40 cycles at 94 °C for 30 s, 60 °C for 30 s, 72 °C for 30 s; 1 cycle at 72 °C for 5 min) and a nested PCR (1 cycle at 94 °C for 15 min; 30 cycles at 94 °C for 30 s, 62 °C for 30 s, 72 °C for 30 s; 1 cycle at 72 °C for 5 min). The amplicons were run on 2% agarose gel, at 110 V for 90 min. The gel was thereafter visualized under Alliance 4.7 transilluminator (UVITEC Limited, Cambridge, UK). 

### 3.4. GenoType^®^ MTBDRplus Assay

Drug susceptibility testing and identification of mutations conferring resistance to RIF and INH was performed using the GenoType^®^ MTBDR*plus* kit (Hain Lifescience, Nehren, Germany), according to the manufacturer’s instructions. Briefly, 5 µL of DNA was amplified with hot-start *Taq* DNA polymerase (Qiagen, Pretoria, South Africa) using biotinylated primers provided in the kit. Amplification was performed using thermal cycler MyCycler^TM^ (Bio-Rad, Cape Town, South Africa). The protocol consisted of 1 cycle at 95 °C for 15 min (*Taq* activation cycle), 10 cycles of denaturation at 95 °C for 30 s and primer annealing at 58 °C for 2 min, 40 cycles of denaturation at 95 °C for 25 s, primer annealing at 53 °C for 40 s and extension at 70 °C for 40 s, followed by a 1 cycle of final extension at 70 °C for 8 min. Subsequent hybridization steps were performed using hybridization trays (Hain Lifescience, Germany) according to the manufacturer’s instructions. Eight *rpoB* wild-type probes (WT1-WT8) and 4 mutant probes (MUT1, MUT2A, MUT2B and MUT3) were used for detecting RIF resistance. One *katG* wild-type (*katG* WT) and 2 mutant probes (MUT1 and MUT2); plus 2 *inhA* wild-type (WT1 and WT2) and 4 mutant probes (MUT1, MUT2, MUT3A and MUT3B) were used for detecting INH resistance. When all WT probes stained positive and no mutation band formed, the result was interpreted as susceptible to the respective antibiotic. The absence of a band for at least one of the WT probes indicated resistance to the respective antibiotic, according to the manufacturer’s instructions.

## 4. Conclusions

The results obtained from our study revealed the presence of MTBC and a high prevalence of MDR in dairy milk in the Nkonkobe region. This highlights the need for villagers to avoid consuming unpasteurized milk due to the potential of zoonotic transmission.

## References

[1] Du Y., Qi Y., Yu L., Lin J., Liu S., Ni H., Pang H., Liu H., Si W., Zhao H., Wang C. (2011). Molecular characterization of *Mycobacterium tuberculosis* complex (MTBC) isolated from cattle in northeast and northwest China. Res. Vet. Sci..

[2] Michel A.L., Hlokwe T.M., Coetzee M.L., Mare L., Connoway L., Rutten V.P.M.G., Kremer K. (2008). High *Mycobacterium bovis* genetic diversity in a low prevalence setting. Vet. Microbiol..

[3] Hlokwe T.M., Jenkins A.O., Streicher E.M., Venter E.H., Cooper D., Godfroid J., Michel A.L. (2011). Molecular characterisation of *Mycobacterium bovis* isolated from African buffaloes (*Syncerus caffer*) in Hluhluwe-iMfolozi Park in KwaZulu-Natal, South Africa. Onderstepoort. J. Vet. Res..

[4] Michel A.L., Bengis R.G., Keet D.F., Hofmeyr M., de Klerk L.M., Cross P.C., Jolles A.E., Cooper D., Whyte I.J., Buss P., Godfroid J. (2006). Wildlife tuberculosis in South African conservation areas: Implications and challenges. Vet. Microbiol..

[5] Lyashchenko K., Whelan A.O., Greenwald R., Pollock J.M., Andersen P., Hewinson R.G., Vordermeier H.M. (2004). Association of tuberculin-boosted antibody responses with pathology and cell-mediated immunity in cattle vaccinated with *Mycobacterium bovis* BCG and infected with *M. bovis*. Infect. Immun..

[6] Angela D.P., Giuseppina C., Tony F.V., Bijo B., Fatmira S., Giuseppina T. (2006). Detection of *Mycobacterium tuberculosis* complex in milk using polymerase chain reaction (PCR). Food Control.

[7] Chen Y., Chao Y., Deng Q., Liu T., Xiang J., Chen J., Zhou J., Zhan Z., Kuang Y., Cai H., Chen H., Guo A. (2009). Potential challenges to the stop TB plan for humans in China; Cattle maintain *M. bovis* & *M. tuberculosis*. Tuberculosis.

[8] Jeon B.Y., Kim S.C., Je S., Kwak J., Cho J.E., Woo J.T., Seo S., Shim H.S., Park B.O., Lee S.S., Cho S.N. (2010). Evaluation of enzyme-linked immunosorbent assay using milk samples as a potential screening test of bovine tuberculosis of dairy cows in Korea. Res. Vet. Sci..

[9] Cosivi O., Grange J.M., Daborn C.J., Raviglione M.C., Fujikura T., Cousins D., Robinson R.A., Huchzermeyer H.F.A.K., de Kantor I., Meslin F.X. (1998). Zoonotic tuberculosis due to *Mycobacterium bovis* in developing countries. Emerg. Infect. Dis..

[10] Al-Saqur I.M., Al-Thwani A.N., Al-Attar I.M. (2009). Detection of *Mycobacteria* spp in cow’s milk using conventional methods and PCR. J. Vet. Sci..

[11] Gibson A.L., Hewinson G., Goodchild T., Watt B., Story A., Inwald J., Drobniewski F.A. (2004). Molecular epidemiology of disease due to *Mycobacterium bovis* in humans in the United Kingdom. J. Clin. Microbiol..

[12] Campbell P.J., Morlock G.P., Sikes R.D., Dalton T.L., Metchock B., Starks A.M., Hooks D.P., Cowan L.S., Plikaytis B.B., Posey J.E. (2011). Molecular detection of mutations associated with first and second-line drug resistance compared with conventional drug susceptibility testing of *Mycobacterium tuberculosis*. Antimicrob. Agents. Chemother..

[13] Green E., Obi L., Nchabeleng M., de Villiers B.E., Sein P.P., Letsoalo T., Hoosen A.A., Bessong P.O., Ndip R.N. (2010). Drug-susceptibility patterns of *Mycobacterium tuberculosis* in Mpumalanga Province, South Africa: Possible guiding design of retreatment regimen. J. Health. Popul. Nutr..

[14] Da Silva P.E.A., Palomino J.C. (2011). Molecular basis and mechanisms of drug resistance in *Mycobacterium tuberculosis*: Classical and new drugs. J. Antimicrob. Chemother..

[15] Wilson M.L. (2011). Recent advances in the laboratory detection of *Mycobacterium tuberculosis* complex and drug resistance. Clin. Infect. Dis..

[16] Aslan G., Tezcan S., Serin M.S., Emekdas G. (2008). Genotypic analysis of isoniazid and rifampin resistance in drug resistant clinical *Mycobacterium tuberculosis* complex isolates in southern Turkey. Jpn. J. Infect. Dis..

[17] Mokrousov I., Filliol I., Legrand E., Sola C., Otten T., Vyshnevskaya E., Limeschenko E., Vyshnevskiy B., Narvskaya O., Rastogi N. (2002). Molecular characterization of multiple-drug-resistant *Mycobacterium tuberculosis* isolates from northwestern Russia and analysis of rifampin resistance using RNA/RNA mismatch analysis as compared to the line probe assay and sequencing of the *rpoB* gene. Res. Microbiol..

[18] Lacoma A., Sierra N.G., Prat C., Manzano J.R., Haba L., Roses S., Maldonado J., Dominguez J. (2008). GenoType MTBDR*plus* assay for molecular detection of rifampin and isoniazid resistance in *Mycobacterium tuberculosis* strains and clinical samples. J. Clin. Microbiol..

[19] Vitale F., Capra G., Maxia L., Reale S., Vesco G., Caracappa S. (1998). Detection of *Mycobacterium tuberculosis* complex in cattle by PCR using milk, lymph node aspirates, and nasal swabs. J. Clin. Microbiol..

[20] Humblet M.F., Boschiroli M.L., Saegerman C. (2009). Classification of worldwide bovine tuberculosis risk factors in cattle: A stratified approach. Vet. Res..

[21] Kazwala R.R., Kambarage D.M., Daborn C.J., Nyange J., Jiwa S.F.H., Sharp J.M. (2001). Risk factors associated with bovine tuberculosis in cattle in the southern highlands of Tanzania. Vet. Res. Commun..

[22] Munyeme M., Muma J.B., Skjerve E., Nambota A.M., Phiri I.G.K., Samui K.L., Dorny P., Tryland M. (2008). Risk factors associated with bovine tuberculosis in traditional cattle of the livestock/wildlife interface areas in the Kafue basin of Zambia. Prev.Vet. Med..

[23] Ameni G., Erkihun A. (2007). Bovine tuberculosis on small-scale dairy farms in Adama Town, central Ethiopia, and farmer awareness of the disease. Rev. Sci. Tech. Off. Int. Epiz..

[24] Ameni G., Vordermeier M., Firdessa R., Aseffa A., Hewinson G., Gordon S.V., Berg S. (2011). *Mycobacterium tuberculosis* infection in grazing cattle in central Ethiopia. Vet. J..

[25] Srivastava K., Chauhan D.S., Gupta P., Singh H.B., Sharma V.D., Yadav V.S., Sreekumaran P., Thakral S.S., Dharamdheeran J.S., Nigam P., Prasad H.K., Katoch V.M.  (2008). Isolation of *Mycobacterium bovis* & *M. tuberculosis* from cattle of some farms in north India—Possible relevance in human health. Indian J. Med. Res..

[26] Sechi L.A., Zanetti S., Sanguinetti M., Molicotti P., Romano L., Leori G., Delogu G., Boccia S., La Sorda M., Fadda G. (2001). Molecular basis of rifampin and isoniazid resistance in *Mycobacterium bovis* strains isolated in Sardinia, Italy. Antimicrob. Agents. Chemother..

[27] Hofmann-Thiel S., van Ingen J., Feldmann K., Turaev L., Uzakova G.T., Murmusaeva G., van Soolingen D., Hoffmann H. (2009). Mechanisms of heteroresistance to isoniazid and rifampin of *Mycobacterium tuberculosis* in Tashkent, Uzbekistan. Eur. Respir. J..

[28] Bazira J., Asiimwe B.B., Joloba M.L., Bwanga F., Matee M.I. (2010). Use of the GenoType^®^ MTBDR*plus* assay to assess drug resistance of *Mycobacterium tuberculosis* isolates from patients in rural Uganda. BMC Clin. Pathol..

